# Diurnal changes of the oral microbiome in patients with alcohol dependence

**DOI:** 10.3389/fcimb.2022.1068908

**Published:** 2022-12-12

**Authors:** Xiangxue Li, Kangqing Zhao, Jie Chen, Zhaojun Ni, Zhoulong Yu, Lingming Hu, Ying Qin, Jingwen Zhao, Wenjuan Peng, Lin Lu, Xuejiao Gao, Hongqiang Sun

**Affiliations:** ^1^ Peking University Sixth Hospital, Peking University Institute of Mental Health, NHC Key Laboratory of Mental Health (Peking University), National Clinical Research Center for Mental Disorders (Peking University Sixth Hospital), Beijing, China; ^2^ Addiction Medicine Department, The Second People’s Hospital of Guizhou Province, Guizhou, China

**Keywords:** alcohol dependence, diurnal oscillation, oral microbiota, liver injure, KEGG functional pathway

## Abstract

**Background:**

Saliva secretion and oral microbiota change in rhythm with our biological clock. Dysbiosis of the oral microbiome and alcohol consumption have a two-way interactive impact, but little is known about whether the oral microbiome undergoes diurnal changes in composition and function during the daytime in patients with alcohol dependence (AD).

**Methods:**

The impact of alcohol consumption on the diurnal salivary microbiome was examined in a case-control study of 32 AD patients and 21 healthy control (HC) subjects. We tested the changes in microbial composition and individual taxon abundance by 16S rRNA gene sequencing.

**Results:**

The present study is the first report showing that alcohol consumption enhanced the richness of the salivary microbiome and lowered the evenness. The composition of the oral microbiota changed significantly in alcohol-dependent patients. Additionally, certain genera were enriched in the AD group, including *Actinomyces*, *Leptotrichia*, *Sphaerochaeta* and Cyanobacteria, all of which have pathogenic effects on the host. There is a correlation between liver enzymes and oral microbiota. KEGG function analysis also showed obvious alterations during the daytime.

**Conclusion:**

Alcohol drinking influences diurnal changes in the oral microbiota, leading to flora disturbance and related functional impairment. In particular, the diurnal changes of the oral microbiota may open avenues for potential interventions that can relieve the detrimental consequences of AD.

## Graphical Abstract

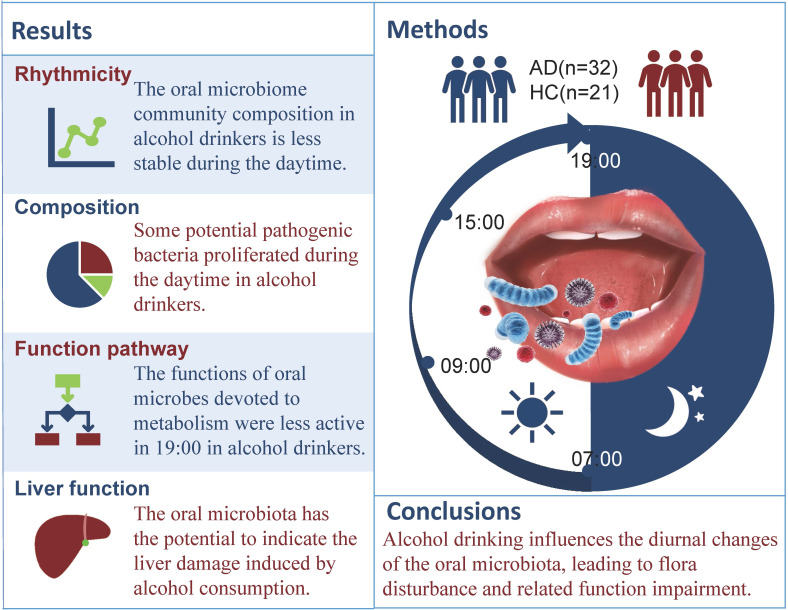

## Introduction

Alcohol dependence (AD) is widespread worldwide (Gilmore et al., 2016). In a current national epidemiological survey comparing drinking prevalence in China, the 12-month prevalence was 1.8%, and the lifetime prevalence was 4.4% (Huang et al., 2019). Alcohol consumption has been linked to many diseases, including liver disease, neuroinflammation, malnutrition, and elevated cancer risk (Schuckit, 2009). Many of these consequences of alcohol consumption have been linked to alcohol-induced microbial dysbiosis (Dubinkina et al., 2017; Fan and Pedersen, 2021), but most previous studies have concentrated on gut microbes. Alcohol use impairs the intestinal barrier and causes changes to the intestinal permeability as well as the gut microbiota composition (Wang et al., 2020), and gut microbiota dysbiosis during chronic alcohol exposure was closely correlated with alcohol-induced neuropsychic behaviors (Xu et al., 2019). Our previous study also revealed the intimate connection between gut microbiota and brain function (Wang et al., 2021). As the beginning of the gastrointestinal tract, the oral cavity, which has been shown to be the second most complex environment in the body after the colon, is gaining more attention (He et al., 2015).

Oral microbes produce nutritional substances (Homer et al., 1990; Jie Bao et al., 2008), including amino acids, peptides and short-chain fatty acids. The changes in the microbiota (i.e., dysbiosis) can alter the function of the community and have a significant impact on health (Orr et al., 2020). Therefore, oral microbes may be an important driving force for some diseases and their related symptoms. Stability is an important trait of the oral microbiome, which has the highest alpha diversities but among the lowest beta diversities compared to other tested body regions (Nasidze et al., 2009). Many factors do not significantly influence the composition of the oral microbiome, including diet, climate, weight, sex, innate genetic variation and geographic location. Meanwhile, only the host species and the related diseases are the primary determinants (Cameron et al., 2015). Therefore, changes in the stability of the oral microbiota are meaningful for detecting body health. Many studies have shown that the oral microbiota has a strong connection with neuropsychiatric disorders, such as autism spectrum disorder (Hicks et al., 2018), Alzheimer’s disease (Ryder, 2020) and cognitive decline in elderly individuals (Vanhatalo et al., 2021). The newly discovered bacterial signature could allow for the deployment and stratification of soldiers with posttraumatic stress disorder according to changes in oral microbiota abundance (Levert-Levitt et al., 2022).

Clock genes show circadian rhythms in salivary glands, and peripheral clock genes can regulate the water channel gene aquaporin-5 and then rhythmically change the type, amount, and content of saliva (Zheng et al., 2012; Papagerakis et al., 2014). These changes in saliva also lead to further changes in the oral microbiota in rhythm. Previous studies have found that the oral microbiome fluctuates over the course of a day (Marsh et al., 2016). For the functional assignment of the annotated genes recognized by the KEGG database, the function of ‘Environmental information processing’ was enriched in the evening, whereas ‘Metabolism’ was enriched in the morning (Takayasu et al., 2017). This rhythmic oral microbiota is important and provides benefits for the host (Marsh et al., 2016), such as preventing colonization of exogenous microorganisms, engaging in cross-talk with the host (Peschel and Sahl, 2006), and regulating proinflammatory responses (Alzahrani et al., 2020).

Chronic alcohol drinking was found to affect the oral mucosa, salivary glands, and saliva (Riedel et al., 2005); therefore, alcohol consumption may influence the amount, composition, and rhythmicity of the oral microbiota. Previous studies proved that alcohol consumption is associated with changes in the oral microbiota, such as a reduced abundance of *Lactobacillales* and elevated abundances of *Neisseria, Streptococcus* and *Prevotella* (Fan et al., 2018b; Barb et al., 2022). Given the abovementioned findings, little is known about whether the oral microbiome varies in composition in the daytime in AD patients and whether it differs from that in HC subjects. We detected the characteristics of the oral microbiome and tested the relationships between alcohol dependence and diurnal changes in the oral microbiome with the oral microbiome at the four time points during the daytime to determine the diurnal changes in the oral microbiome.

## Material and methods

### Subjects

Male patients (n = 32) recruited to this study were between 18 and 60 years of age with alcohol dependence according to the Diagnostic and Statistical Manual of Mental Disorders-IV (DSM-IV), established by the Mini International Neuropsychiatric Interview (MINI). Subjects did not have other substance use disorders or other mental disorders in DSM-IV on Axis I, except nicotine dependence, as determined by the MINI. Patients were admitted to The Second People’s Hospital of Guizhou Province and assessed for self-reported drinking conditions (including daily consumption, age at first drink, drinking years, and withdrawal days). After one week or more, they were in stable condition and submitted to tests about withdrawal syndrome (Revised Clinic Institute Alcohol Withdrawal Syndrome Assessment Scale, CIWA-Ar), alcohol cravings (Visual Analog Scale, VAS; Alcohol Urge Questionnaire, AUQ; Pennsylvania Alcohol Craving Scale, PACS), cognitive function (Montreal Cognitive Assessment, MoCA), sleeping condition (Pittsburg Sleep Quality Index, PSQI) and symptoms of depression and anxiety (Hamilton Depression scale, HAMD; Hamilton Anxiety Scale, HAMA). Some biochemical indices related to alcohol consumption, such as aspartate aminotransferase (AST), alanine aminotransferase (ALT), gamma-glutamyltransferase (GGT), and mean corpuscular volume (MCV), were also measured in the AD group.

HC subjects (n =21) were free of somatic and psychiatric illnesses. HC subjects did not have a current or lifetime history of alcohol use or other substance use disorders. The enrollment and exclusion criteria are shown in the **Supplementary Information**.

This study was approved by the Ethics Committee of Peking University Sixth Hospital. All study participants provided written informed consent.

### Sample collection

On the first admission day (before enrollment), after baseline assessment, the subjects meeting enrollment criteria but not exclusion criteria were divided into two groups: the AD group and the HC group. Fresh unstimulated saliva (1-2 ml) was collected at 7:00, 11:00, 15:00, and 19:00 on the second day after enrollment. Before and during the collection day, subjects were not allowed to brush their teeth and consume any food or drink preceding each collection time point for 1 hour. Samples were frozen in liquid nitrogen for 1 minute and stored in the refrigerator at -80°C. Genomics DNA extraction, library construction, sequencing and bioinformatics analysis are shown in **Supplementary**.

### Library construction

Variable regions V4 of the bacterial 16S rRNA gene were amplified with degenerate PCR primers, 515F (5’-GTGCCAGCMGCCGCGGTAA-3’) and 806R (5’- GGACTACHVGGGTWTCTAAT-3’). Both forward and reverse primers were tagged with Illumina adapter, pad, and linker sequences. PCR enrichment was performed in a 50 μL reaction containing 30 ng template, fusion PCR primer, and PCR master mix. The PCR cycling conditions were as follows: 95°C for 3 minutes; 30 cycles of 95°C for 45 seconds, 56°C for 45 seconds, and 72°C for 45 seconds; and a final extension for 10 minutes at 72°C for 10 minutes. The PCR products were purified using Agencourt AMPure XP beads and eluted in the Elution buffer. Libraries were qualified by the Agilent Technologies 2100 bioanalyzer. The validated libraries were used for sequencing on the Illumina HiSeq 2500 platform (BGI, Shenzhen, China) following the standard pipelines of Illumina, generating 2 × 250 bp paired-end reads.

### Sequencing and bioinformatics analysis

Raw reads were filtered to remove adaptors and low-quality and ambiguous bases, and then paired-end reads were added to tags by the Fast Length Adjustment of Short reads program (FLASH, v1.2.11) to obtain the tags. The tags were clustered into OTUs with a cutoff value of 97% using UPARSE software (v7.0.1090), and chimera sequences were compared with the Gold database using UCHIME (v4.2.40) for detection. Then, OTU representative sequences were taxonomically classified using Ribosomal Database Project (RDP) Classifier v.2.2 with a minimum confidence threshold of 0.6 and trained on the Greengenes database v201305 by QIIME v1.8.0. USEARCH_ global was used to compare all tags back to OTUs to obtain the OTU abundance statistics table of each sample.

Alpha diversity were estimated by MOTHUR (v1.31.2) and beta diversity were estimated by QIIME (v1.8.0) at the OTU level, respectively. The sample clustering was conducted by QIIME (v1.8.0) based on UPGMA. KEGG functions were predicted using PICRUST2 (v2.3.0-b)software. Statistically significant KEGG pathways for each group were additionally identified using LEfSe. Barplots of different classification levels were plotted with R package v3.4.1 and R package “gplots”. Principal component analysis (PCA) of OTUs was performed with the R package “ade4”. Partial least-squares discrimination analysis (PLS-DA) was performed by the R package mixOmics. Principal coordinate analysis (PCoA) was performed by QIIME (v1.8.0). LEfSe cluster was conducted by LEfSe. Nonmetric multidimensional scaling ordination (NMDS) was performed by R package. Significant species or functions were determined by R (v3.4.1) based on the Wilcoxon test or Kruskal-test.

### Statistical analysis

Differences in education years, body mass index (BMI), and MoCA scores (continuous variables consistent with normal distribution and homogeneity of variance) between the AD and HC groups were assessed with a two-tailed Student’s t test, with significant differences defined by an uncorrected p < 0.05, and the results are expressed as the mean ± standard deviation. Other continuous variables were expressed as the median (Q25, Q75) and analyzed by a nonparametric test. The discontinuous variables were analyzed by the chi-square test. The alpha diversity index and beta diversity index were calculated from the taxonomic profiles and compared among eight groups (AD-7:00, AD-11:00, AD-15:00, AD-19:00, HC-7:00, HC-11:00, HC-15:00 and HC-19:00). A bar plot was used to represent the composition at the phylum and genus levels. Differential taxon expression of different time points in the AD or HC group was visualized with a multivariate partial least squares discriminant analysis (PLS-DA), and variable importance in projection was determined for each taxon. Differences in individual taxa between 2 groups at different time points were evaluated using a one-way analysis of variance with a false discovery rate (FDR) correction for multiple testing. Significant species or functions were determined by R (v3.4.1) based on the Wilcoxon test or Kruskal-test. Some individual taxa differences among the two groups or four time point groups were investigated with nonparametric Kruskal–Wallis testing, followed by *post hoc* between-group comparisons (7:00 vs. 11:00, 7:00 vs. 15:00, 7:00 vs. 19:00, 11:00 vs. 15:00, 11:00 vs. 19:00, 15:00 vs. 19:00) with a Wilcoxon test. Spearman correlation analysis was performed between biochemical indices and oral microflora of alcohol-dependent patients under different classification levels. P < 0.05 was considered statistically significant. The analysis software used was SPSS 23.0 (SPSS, Chicago, IL, USA).

## Results

### Participant characteristics

A total of 32 AD patients and 21 HC subjects were enrolled and investigated. There was no significant difference between the two groups in terms of age, occupation, education years, marital status, BMI, or FTND (**Table 1**). However, in the AD group, the HAMD, HAMA, and PSQI scores were higher than those in the HC group, while the MoCA score was lower. In all, 30 of the patients (93.75%) smoked cigarettes, while 16 of the healthy controls (76.19%) smoked cigarettes; there was no significant difference between the two groups. In the AD group, the VAS score was 0.5 (0.00, 5.00), the PACS score was 5.5 (3.00, 9.00), the AUQ score was 9 (8.00, 12.00) and the CIWA-Ar score was 17.56 ± 7.90. Specific baseline characteristics by group are shown in **Table 1**.

**Table 1:** Demographic and clinical characteristics of subjects.

	AD (n=32)	HC (n=21)	χ^2^/z/t	p
**Age, year**	43.5 (38.5, 49.5)	42.5 (26.0, 51.0)	-0.98	0.33
**Occupation**	/	/	0.08	0.77
** Employed**	24 (75.0%)	15 (71.4%)	/	/
** Unemployed**	8 (25%)	6 (28.6%)	/	/
**Education Years**	7.46 ± 4.28	8.40 ± 4.12	-0.91	0.37
**Marital Status**	/	/	1.29	0.26
** Married**	27 (84.4%)	15 (71.4%)	/	/
** Single/Divorced/Widowed**	5 (15.6%)	6 (28.6%)	/	/
**Smoking Status**	/	/	/	0.10
** Smoking**	30 (93.75%)	16 (76.19%)	/	/
** Nonsmoking**	2 (6.25%)	5 (23.81%)	/	/
**BMI, kg/m^2^**	21.70 ± 2.32	23.00 ± 3.09	-1.56	0.12
**MoCA**	19.56 ± 6.39	23.79 ± 5.41	-2.41	0.02
**FTND**	4.00 (2.00, 6.00)	4.00 (0.00, 6.00)	-0.69	0.49
**HAMA**	7.00 (2.25, 10.75)	1.00(0.25, 2.75)	-4.36	<0.01
**HAMD**	7.00 (1.25, 11.00)	1.00 (0.00, 2.00)	-3.71	<0.01
**PSQI**	7.50 (4.00, 15.75)	2.50 (1.00, 4.00)	-3.76	<0.01
**Withdrawal Days**	2.00 (1.00, 4.00)	/	/	/
**Drinking Years**	10.00 (5.00, 17.00)	/	/	/
**Age at First Drink**	16.00 (15.00, 18.00)	/	/	/
**Daily Consumption, standard drinks/day**	15.00 (7.00, 23.00)	/	/	/
**CIWA-Ar**	17.56 ± 7.90	/	/	/
**VAS**	0.50 (0.00, 5.00)	/	/	/
**PACS**	5.50 (3.00, 9.00)	/	/	/
**AUQ**	9.00 (8.00, 12.00)	/	/	/

AD, alcohol dependence; AUQ, Alcohol Urge Questionnaire; BMI, body mass index; CIWA-Ar, FTND, HAMA, Hamilton Anxiety Scale; HAMD, Hamilton Depression scale; HC, healthy control; MoCA, Montreal Cognitive Assessment; PACS, Pennsylvania Alcohol Craving Scale; PSQI, Pittsburgh Sleep Quality Index; VAS, Visual Analog Scale.

### Diversity changes

**Figure 1:**
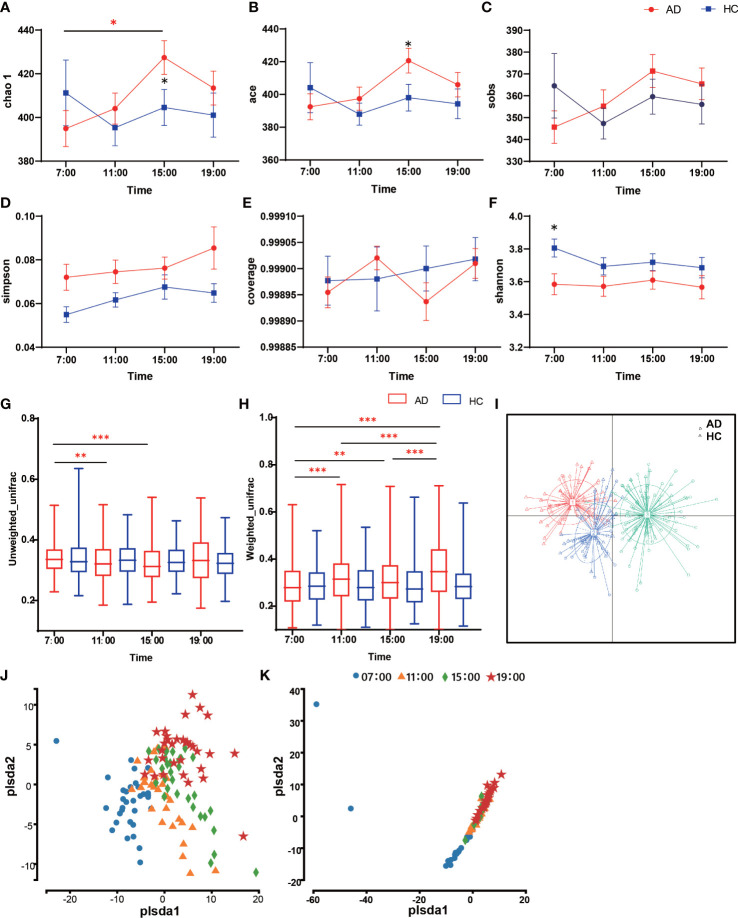
Diversity of salivary microbiota in the AD and HC groups. **(A-F)** The differences in α-diversity in salivary microbiota between AD and HC conditions at each time point (A. Chao; **(B)** ACE; **(C)** Sobs; **(D)** Shannon; **(E)** Good coverage; **(F)** Simpson). **(G, H)** Boxplots of β-diversity differences in salivary microbiota between AD and HC conditions at each time point **(G)**. Unweighted UniFrac; **(H)** Weighted UniFrac). **(I)** Principal coordinate analysis (PCoA) of samples using Jensen-Shannon divergence (JSD). **(J, I)** Partial least squares discrimination analysis (PLS-DA). Points of different colors or shapes represent samples at different time points and in different groups. The scales of the horizontal and vertical axes are relative distances, which have no practical significance. The horizontal and vertical axes represent the suspected influencing factors of the microbial composition differences in the AD group **(J)** and HC group **(K)**, respectively. *Wilcox test indicated significant differences between different groups at one time point, Kruskal-Wallis test indicated significant differences among four time points in either the AD or HC group. All p values are Bonferroni adjusted. * indicates p < 0.05, ** indicates p < 0.01, *** indicates p < 0.001. Data are expressed as the mean ± SEM (n=21-32).

In the α-diversity analysis, the Chao 1 and ACE indices at 15:00 increased in the AD group with statistical significance relative to the HC group (Chao 1 Z = -2.04, p = 0.042; ACE Z = -2.02, p = 0.044; **Figures 1A, B**). Within the AD group, there was a significant difference between the 7:00 and 15:00 Chao indices (χ^2^ = 8.65, p = 0.034). However, the observed species (Sobs), Simpson, and Coverage indices showed no significant differences (p > 0.05; **Figures 1C-E**). The Shannon indices in the AD group were higher than those in the HC group in general, but the differences were not statistically significant except at 7:00 (Z = 2.13, p = 0.033; **Figure 1F**). When assessing β-diversity, we found that the oral microbiota fluctuated during the daytime according to the unweighted UniFrac distance in the AD group, while in the HC group, it showed nearly no changes (p > 0.05; **Figure 1G**). At 7:00, the AD group had the highest unweighted UniFrac diversity, with a significant difference compared with 11:00 and 15:00 (χ^2^ = 24.23, p < 0.001; *post hoc* 7:00 vs. 11:00 p = 0.007; 7:00 vs. 15:00 p < 0.001). The Weighted UniFrac distance showed the same pattern: the HC group did not change, while the AD group had a significant diurnal variation except at 11:00 and 15:00 (χ^2^ = 77.68, p< 0.000; *post hoc* 7:00 vs. 11:00 p < 0.001; 7:00 vs. 15:00 p = 0.001; 7:00 vs. 19:00 p < 0.001; 11:00 vs. 19:00 p < 0.001; 15:00 vs. 19:00 p < 0.001; 11:00 vs. 15:00 p > 0.05; **Figure 1H**). Principal coordinate analysis (PCoA) of samples using a Jensen-Shannon divergence (JSD) showed that the samples were clustered into 3 groups (**Figure 1I**). In partial least squares discrimination analysis (PLS-DA), all samples from four different time points were gathered into 4 clusters in the AD group (**Figure 1J**), but in the HC group, the microbiomes of the four time points clustered together (**Figure 1K**). We also performed ANOISM and found that the difference at the four time points was not as obvious (AD r = 0.075, p < 0.001; HC r=0.072, p < 0.01; **Supplementary Figure 1**).

### Microbial differences

**Figure 2:**
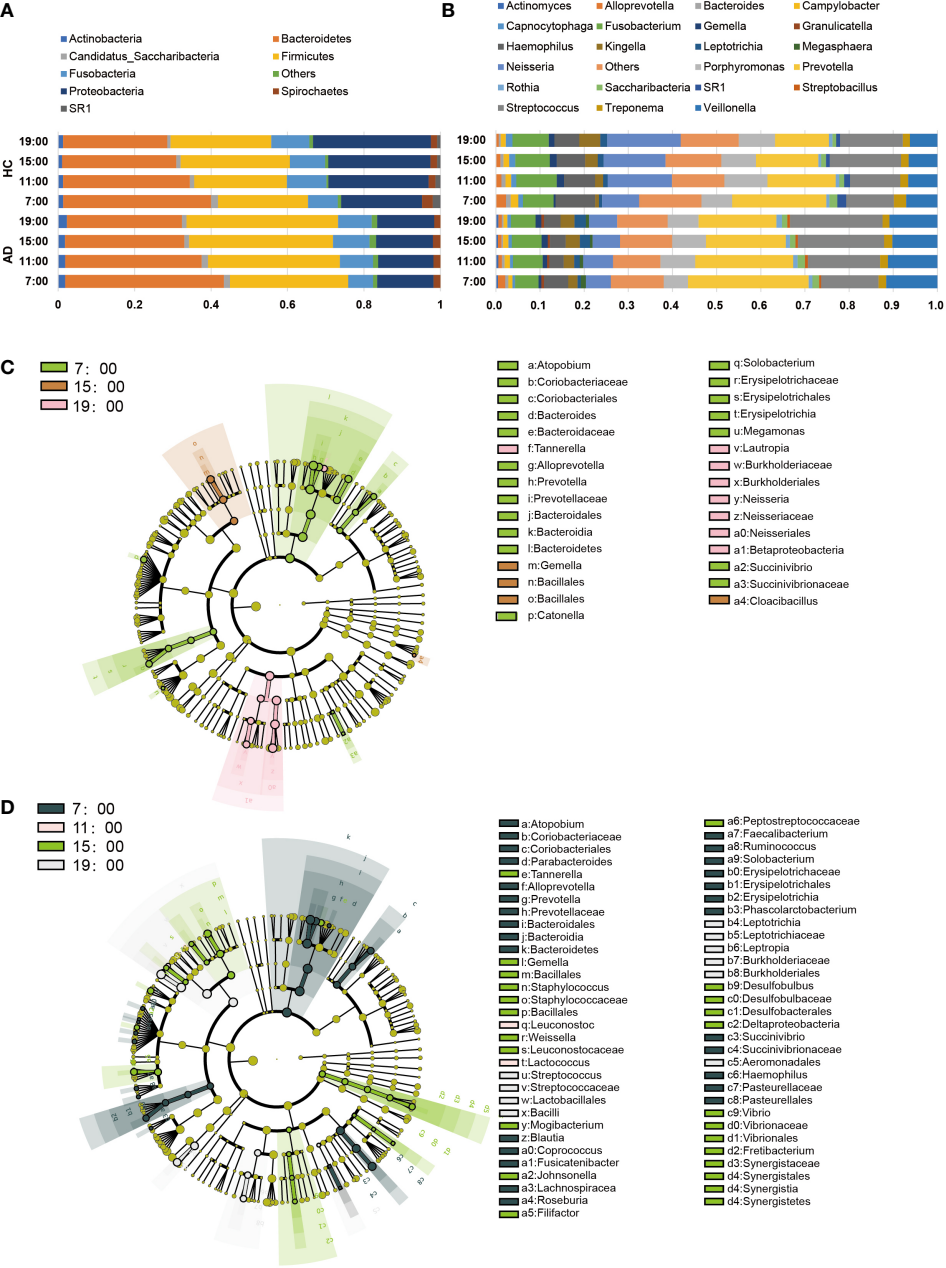
Changes in the core salivary microbiome. **(A, B)** Each horizontal bar represents the relative abundances in the saliva sample at different times and in different groups **(A)**. phylum level; **(B)** genus level). **(C, D)** LEfSe Cluster. Different colors represent different groups. Nodes with different colors represent the microflora that play an important role in the group represented by this color. A colored dot represents a biomarker, and the name of the biomarker is shown in the upper right legend. The yellow nodes represent groups of microbes that do not play an important role in the different groups. From inside to outside, each circle is the species at the level of phylum, class, order, family, and genus. **(C)** LEfSe of the HC group, **(D)** LEfSe of the AD group.

**Figure 3:**
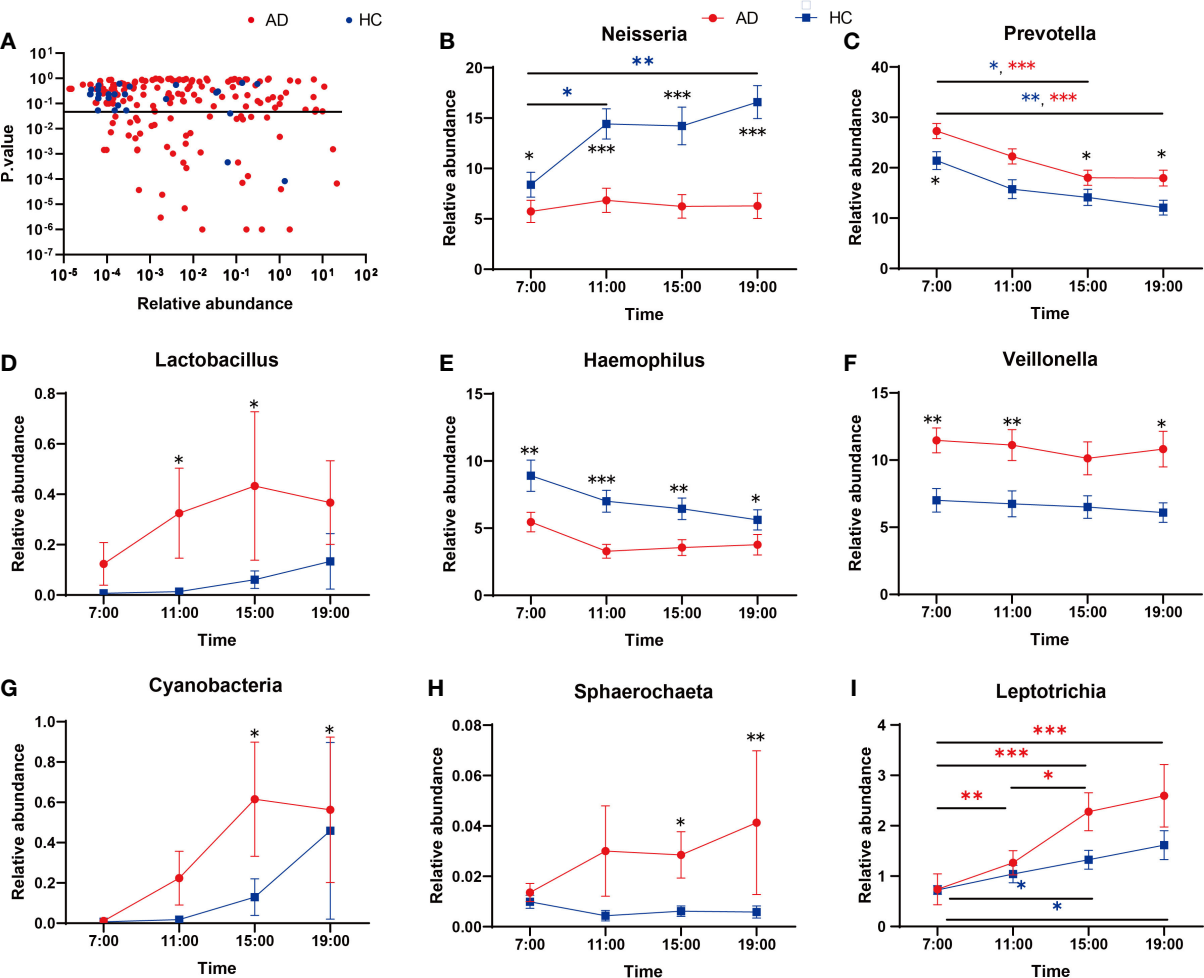
**(A)** Genera showing variation at different time points. The Kruskal-Wallis test indicated significant differences between time points in both AD and HC. The dashed line indicates p = 0.05. **(B-I)** Relative abundances of significant taxa in AD and HC conditions across time points: **(B)** *Neisseria*, **(C)** *Prevotella*, **(D)** *Lactobacillus*, **(E)** *Haemophilus*, **(F)** *Veillonella*, **(G)** *Cyanobacteria*, **(H)** *Sphaerochaeta*, **(I)** *Leptotrichia*. *Wilcox Test indicated significant differences between different groups at one time point, Kruskal-Wallis test indicated significant differences between time points in either AD and HC. All p values are Bonferroni adjusted. * indicates p < 0.05, ** indicates p < 0.01, *** indicates p < 0.01. Data are expressed as the mean ± SEM (n=21-32).

Regarding the abundance of specific oral microbiota taxa, at the phylum level, Bacteroidetes decreased in the daytime in both the AD and HC groups (AD χ^2^ = 31.12, p < 0.001; HC χ^2^ = 19.26, p < 0.001), while Firmicutes increased only in the AD group (AD χ^2^ = 10.89, p = 0.012; HC p > 0.05; **Figure 2A**). The HC group had a richer abundance of Proteobacteria and increased in the daytime, but it did not change with a significant difference in the AD group (AD p > 0.05; HC χ^2^ = 9.20, p = 0.027; **Supplementary Figures 2A-D**). Alcohol consumption was a significant factor for some individual bacteria (relative abundances) at the taxonomic levels of phyla and genera (**Figure 2B**). LEfSe was used for the analysis of different species among the groups at four time points (**Figures 2C, D**). Different quantities of genera with significant differences (FDR < 0.05) were shown at different time points between the AD and HC groups: 32 genera at 7:00, 23 genera at 11:00, 28 genera at 15:00, and 29 genera at 19:00 (**Supplementary Figures 3A-D**). There were more microbiota with diurnal changes in the AD group at the phylum level, such as Bacteroidetes, Firmicutes, Fusobacteria, Cyanobacteria, Synergistetes, and Elusimicrobia. Forty-six bacterial changes occurred in the daytime at the genus level, including *Leptotrichia, Prevotella*, and *Lactococcus*. In the HC group, bacteria with diurnal changes at the phylum level included Bacteroidetes, Proteobacteria, Cyanobacteria, and Acidobacteria; 23 bacteria at the genus level were altered in the daytime, including *Prevotella, Lactococcus*, and Neisseria (**Figure 3A**). The abundances of some bacteria changed considerably in the AD group compared with the HC group, including *Neisseria, Prevotella, Lactobacillus, Haemophilus, Veillonella*, *Sphaerochaeta, Leptotrichia* and Cyanobacteria. (**Figures 3B-I**). In addition, only the abundances of *Neisseria* and *Haemophilus* were richer in the HC group with diurnal changes, while the rest of the genera mentioned above were all richer in the AD group.

### Functional analysis

**Figure 4:**
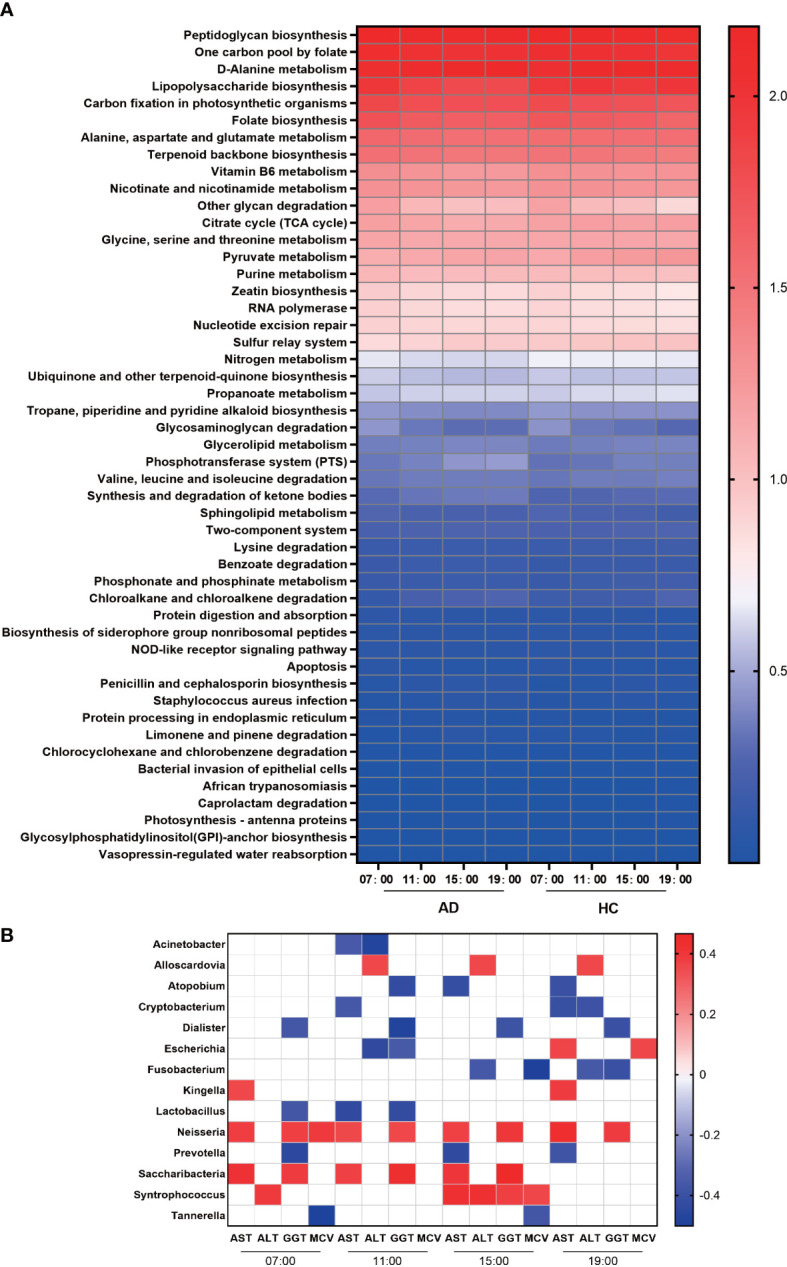
Function and correlation analysis. **(A)** Heatmap of the abundances of KEGG pathways in the AD and HC groups during the daytime. **(B)** Heatmap of Spearman’s rank correlation coefficients of different genera and biochemical indices of liver damage at different times. Those without associations were deleted and are not shown in this figure. White squares indicate that the correlations were not statistically significant. p < 0.05 and permutation p < 0.05 for all data selected.

We used PICRUSt to assess the functional content of the microbiota based on the 16S data and found a total of 408 KEGG modules by using the KEGG database. The results detected 68 modules that exhibited significant differences during the daytime in the AD group and 43 in the HC group at level 3. At 07:00, many metabolic pathways, such as “lipopolysaccharide biosynthesis”, “one-carbon pool by folate”, “carbon fixation in photosynthetic organisms”, “folate biosynthesis”, and “glycosaminoglycan degradation”, were active in both the AD and HC groups, and at 19:00, “phosphonate and phosphinate metabolism”, “chloroalkane and chloroalkene degradation”, and “pyruvate metabolism” were enriched. In addition, “genetic information processing” and “cellular processes” were enriched at 07:00; the former included “mismatch repair”, “nucleotide excision repair”, “RNA polymerase” and “ribosome”, and the latter included “cell cycle Caulobacter” and “apoptosis”. In the HC group, the metabolic function module was active at 19:00 and involved lipid metabolism: “fatty acid biosynthesis”, “glycerophospholipid metabolism”, and “glycerolipid metabolism”; energy metabolism: “tyrosine metabolism” and “taurine and hypotaurine metabolism”; and amino acid metabolism: “valine, leucine, and isoleucine degradation”, “tryptophan metabolism”, and “glutathione metabolism”. The 3 metabolic pathways, “valine, leucine and isoleucine degradation”, “propanoate metabolism”, and “benzoate degradation”, were active in the HC group at 19:00, but in the AD group, they were enriched at 11:00 and 15:00 (**Figure 4A**, **Supplementary Figures 4A, B**).

### Relationships between the oral microbiome and biochemical indices of liver impairment

In the AD group, we analyzed the correlations between the relative abundances of different genera and biochemical indices at the four time points. We found that the abundances of *Dialister, Lactobacillus, Escherichia, Atopobium, and Prevotella* were negatively associated with the levels of GGT, while the abundances of *Leptotrichia, Neisseria, Saccharibacteria*, and *Syntrophococcus* were positively associated with GGT. Many bacteria were positively associated with AST, including *Kingella, Neisseria, Saccharibacteria, and Wolinella*, and only *Prevotella* and *Lactobacillus* were negatively associated. ALT was correlated with the abundances of *Parascardovia, Escherichia, Fusobacterium, Klebsiella* and so on. Regarding MCV, *Neisseria, Escherichia*, and *Syntrophococcus* were positively associated, and *Tannerella* and *Fusobacterium* were negatively related (**Figure 4B**).

## Discussion

This is the first study to measure the microbiome abundance at four time points during the daytime for AD and HC groups and determine the changes between the two groups and their four time points. We found that (a) the oral microbiome community composition in the AD group was less stable during the daytime than that in the HC group. (b) Some potential pathogenic bacteria proliferated during the daytime in alcohol drinkers. However, the abundance of *Neisseria* decreased in the AD group, which is inconsistent with a previous study (Fan et al., 2018b). (c) Alcohol dependence may be an important influencing factor on oral microbial patterns (stomatotypes) (Willis et al., 2018). (d) The functions of oral microbes devoted to metabolism were active at 7:00 and 19:00 in the HC group, but the AD group had less activity in metabolic pathways at 19:00. (e) The oral microbiota has the potential to indicate the liver damage induced by alcohol consumption. These results suggest that alcohol drinking influences the diurnal changes of the oral microbiota, leading to flora disturbance and related functional impairment.

In the diversity analysis (**Figure 1**), we found that alcohol consumption enhanced the richness of the oral microbiome and lowered the evenness, as shown in a previous study (Fan et al., 2018a). The PLS-DA analysis found that the oral microbiome in alcohol-dependent patients changed significantly during the daytime. The results inspired us to conclude that research on the oral microbiota of alcohol-dependent patients should restrict the time of sample collection.

When talking about specific bacteria, we found that the AD group had more taxa with significant diurnal rhythmicity at the same time (**Figure 2**). The oral bacterial community compositions of all the subjects were analyzed at different taxonomic levels. At the phylum level, the abundance of Bacteroidetes gradually decreased during the daytime in both groups with a significant difference, while Firmicutes increased with a significant difference in the AD group, but its growth was not evident in the HC group (**Supplementary Figures 2A, B**). As two major phyla in the human gut microbiota, changes in the abundances of Bacteroidetes and Firmicutes are related to cancer (Flemer et al., 2017), obesity (Magne et al., 2020) and Alzheimer’s disease (Vogt et al., 2017). However, we still do not know the meaning of the diurnal changes of the two phyla, which are also the major phyla comprising the oral microbiome.

At the genus taxonomic level, the abundance of *Neisseria* increased in the HC group but did not change in the AD group during the day (**Figure 3B**). *Neisseria* has high ADH and low ALDH activities and produces significant amounts of acetaldehyde when cultured in a medium containing ethanol *in vitro* (Kurkivuori et al., 2007). However, in our study, the abundance of *Neisseria* was lower in the AD group than in the HC group. The discrepancy probably lies in some differences from the previous study (Fan et al., 2018b): most of the subjects in our research had a smoking habit, while theirs excluded smokers, as smoking might eradicate *Neisseria* in oral microflora, as reported previously (Muto et al., 2000). *Neisseria* is less abundant in current smokers than in nonsmokers (Sato et al., 2020), and univariate analysis for the different variables reveals that smoking is the most important factor that contributes significantly to increased salivary acetaldehyde production from ethanol (Kurkivuori et al., 2007). Then, we performed two-way ANOVA and found no evidence showing that smoking had any impact on Neisseria (**Supplementary Table 1**). Most of the AD patients we recruited had been drinking for more than 10 years; our bodies have mechanisms of adaptive regulation, meaning that the abundance of *Neisseria* and the activity of ADH decrease to prevent excessive acetaldehyde production. After all, the metabolism of ethanol to acetaldehyde or acetate is associated with the production of reactive oxygen species and oxidation of the fatty acids in phospholipids, which is associated with neurotoxicity and neurodegeneration (Hernandez et al., 2016).

*Prevotella* was another important genus whose abundance changed during the daytime and decreased gradually in both the AD and HC groups (**Figure 3C**). *Prevotella* is involved in the pathogenesis of periodontitis (Ibrahim et al., 2017); other periodontitis-related bacteria were all increased in the AD group, including *Actinomyces* (Vielkind et al., 2015) and *Lactobacillales* (Caufield et al., 2015) (**Figure 3D**). Alcohol consumption is related to moderately increased severity of periodontal disease (Tezal et al., 2001), and many oral microbes are related to inflammation pathways (Mei et al., 2020). A previous study showed that alcohol consumption can enhance oral inflammation (Irie et al., 2008); therefore, our results enlighten us that oral microbiota can be an intermediary for alcohol to induce oral immunoreaction.

In the AD group, the abundance of Cyanobacteria increased gradually from 7:00 and peaked at 15:00, while that in the HC group fluctuated less overall (**Figure 3G**). Cyanobacteria can produce a wide range of secondary metabolites, including multiple toxins targeting the liver, nervous system, skin, and gastrointestinal tract (Buratti et al., 2017). The cyanobacteria neurotoxin β-N-methylamino-L-alanine is bound to protein and functions as an endogenous neurotoxic reservoir (Murch et al., 2004), which may help explain some nervous system symptoms in AD patients. In addition, the relative abundances of *Leptotrichia* and *Sphaerochaeta* in the saliva of patients with cognitive decline induced by Alzheimer’s disease greatly increased (Liu et al., 2019); our research also illustrated the increases in these two genera in the AD group with different degrees of cognitive impairment.

*Neisseria, Haemophilus, Porphyromonas, Prevotella*, and *Veillonella* are the most abundant genera in the oral microbiome. In some studies, the oral microbiome samples were clustered into two broad compositional patterns (also named stomatotypes) (**Figures 3B–E**). They defined higher proportions of *Neisseria, Haemophilus*, and *Porphyromonas* as one type (stomatotype 1, coinhabiting Group 2) and those of *Prevotella* and *Veillonella* in the other (stomatotype 2, coinhabiting Group 1) (Takeshita et al., 2016; Willis et al., 2018). These two stomatotypes represent two steady equilibria in the oral microbiome that reflect the potential restriction of the human oral niche and are not influenced by geographical regions, lifestyles, and ages. However, in our research, the HC group had higher proportions of *Neisseria and Haemophilus*, close to stomatotype 1, and the AD group had more *Prevotella* and *Veillonella*, close to stomatotype 2. Therefore, alcohol dependence may be an influencing factor on oral microbial patterns.

The circadian oscillation of gene functions was also evaluated. Overall, the HC group was richer than the AD group in many functions, such as ‘Glycan biosynthesis and metabolism’, ‘Glycan biosynthesis and metabolism’, ‘Endocrine system’ and ‘Immune diseases’ (**Figure 4A**). Protein and glycoprotein components of saliva play a particularly important role in modulating the oral microbiota and helping with the clearance of pathogens (Cross and Ruhl, 2018), so the decrease in ‘Glycan biosynthesis and metabolism’ could impair the host defense mechanisms in the oral cavity. Varying degrees of the decrease in other functions also implied that alcohol consumption could influence the physiological function of the human oral microbiome. In addition, morning and evening are important times for bacterial metabolism. Many important metabolic pathways were enriched significantly at 7:00 and 19:00. In the HC group, metabolic function modules were active at 19:00, including ‘lipid metabolism’, ‘energy metabolism’, and ‘amino acid metabolism’. However, in the AD group, there were few active metabolic pathways at 19:00. A previous study proved that these functional oscillations may be related to the maintenance of oral homeostasis (Takayasu et al., 2017). We found that the functions of oral microbes devoted to metabolism were active at 7:00 and 19:00 in the HC group, but the AD group had less activity in metabolic pathways at 19:00, so alcohol consumption may lead to metabolic disorders of the oral microbiota.

Serum ALT and AST levels and their ratio (AST/ALT ratio) are commonly measured clinically as biomarkers for liver health (Dufour et al., 2000) and alcohol-associated liver disease (Andresen-Streichert et al., 2018). Significant correlations have been found between daily alcohol intake and corresponding GGT and MCV values (Papoz et al., 1981) (**Figure 4B**). Some bacteria only had correlations with the levels of serum biochemical indices at specific times. However, *Neisseria* had strong relationships with AST and GGT at all four time points. Previous studies have confirmed that *Neisseria* showed higher relative abundance in primary biliary cirrhosis patients than in healthy controls (Lv et al., 2016). In the oropharynx, the abundance of *Neisseria* is higher in HBV-decompensated cirrhosis patients with confirmed pneumonia than in HBV-decompensated cirrhosis patients without pneumonia and healthy subjects (Lu et al., 2015). *Neisseria* may be involved in orchestrating inflammatory disease by promoting inflammation and remodeling normally benign microbiota into a dysbiosis community (Chen et al., 2016). Therefore, oral microbiota may have the potential to indicate the liver damage induced by alcohol consumption.

There are still some limitations in our study. (a) Saliva is easy to collect and contains an abundant oral microbiome, but there are many periodontal bacteria that exist within the subgingival niche or dental plaque (Filoche et al., 2010). Therefore, more samples from other parts of the oral cavity should be included in future analyses. (b) We discovered that the compositions and rhythms of many bacteria were significantly altered in the AD group during the daytime, but whether these changes promote the development of AD and the mechanisms involved require further research. (c) We only collected samples at 12 hours at the four time points from each subject, so we cannot deduce the circadian rhythm of the oral microbiome and its relationship with central and peripheral clock genes. (d) Because the prevalence of AD in males is 35 times greater than that in females (Huang et al., 2019), we only collected saliva samples from male AD patients, and the results can only represent the majority of patients.

In summary, we are the first to compare the diurnal changes in the oral microbiota in AD patients and healthy subjects. Our study finds that the oral microbiome in AD patients is more unstable during the daytime than that in HC, and the composition of the oral microbiota was altered in AD patients during the daytime, especially some potential pathogenic bacteria that proliferate during the daytime in alcohol drinkers, providing further support for the influence of alcohol consumption on the oral microbiome and the role of the oral microbiome in AD pathogenesis. Meanwhile, some functions of the oral microbiota were also impaired in the AD group, which is related to the maintenance of oral homeostasis. Moreover, alcohol dependence may be an important influencing factor on the ‘stomatotypes’, and the composition of the oral microbiota differs in AD patients with damage to liver function. Therefore, diurnal changes in the oral microbiota may be a potential biomarker for AD.

## Data availability statement

The data presented in the study are deposited in the SRA repository, accession number: PRJNA895096.

## Ethics statement

The studies involving human participants were reviewed and approved by Ethics Committee of Peking University Sixth Hospital. The patients/participants provided their written informed consent to participate in this study.

## Author contributions

XL analyzed the study data and wrote the initial draft (including substantive translation). KZ conducted the research and investigations, specifically performing the experiments and data collection. ZN, ZY, LH, JC, LL, XG and HS revised the manuscript and participated in editing, interpretation, and revision. All authors contributed to the article and approved the submitted version.
