# Supplementary figures and images for: Diurnal changes of the oral microbiome in patients with alcohol dependence
Source: Front Cell Infect Microbiol. 2022 Dec 12;12:1068908. doi: 10.3389/fcimb.2022.1068908 (PMC9791055; doi:10.3389/fcimb.2022.1068908)

A

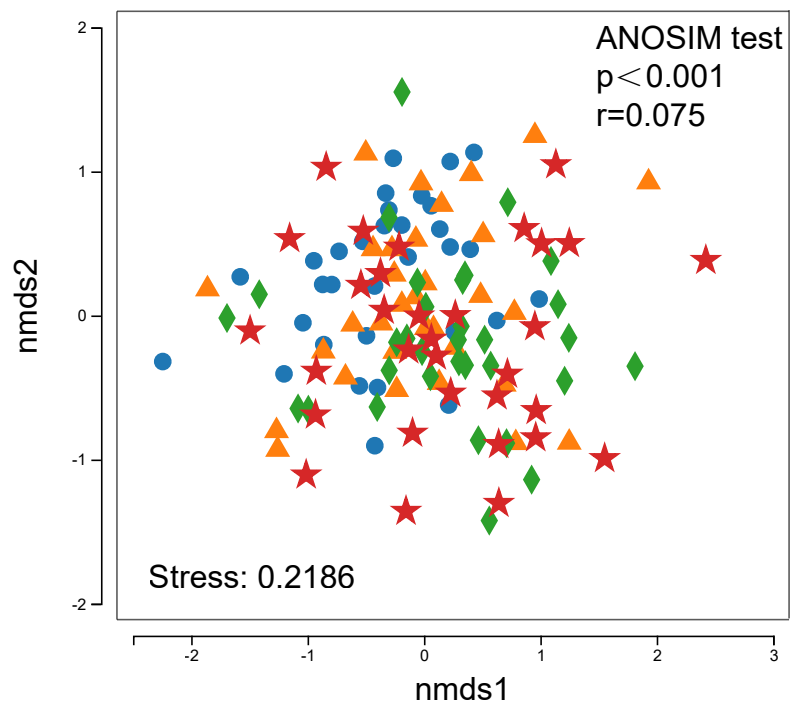

B

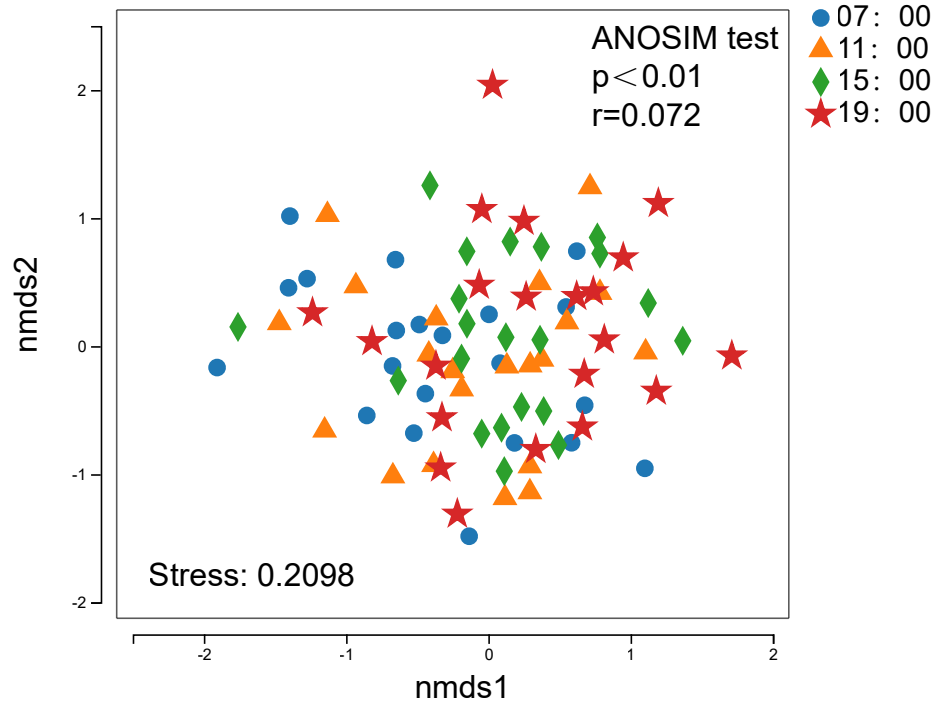

Supplement: Supplementary file 1 [file DataSheet_1.pdf]

**A****Firmicutes**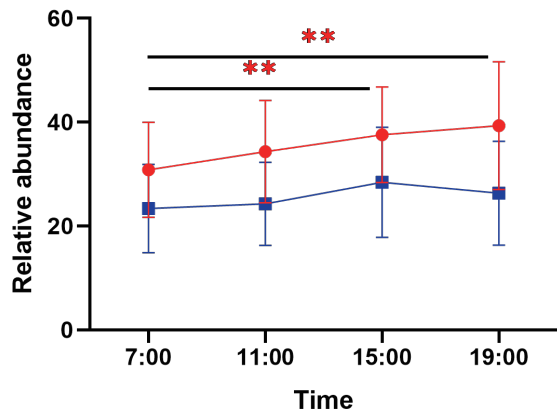**B****Bacteroidetes**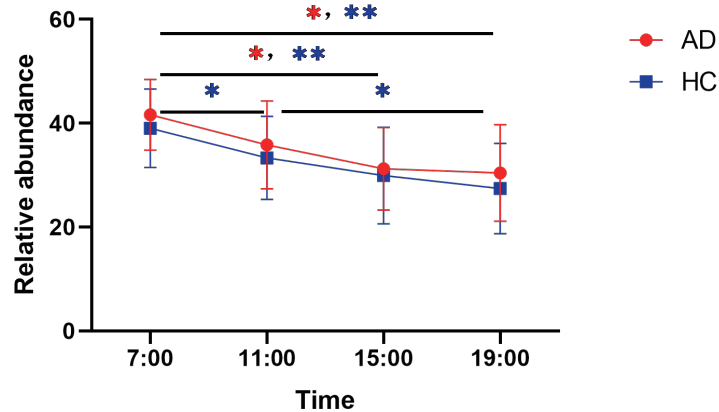**C****Proteobacteria**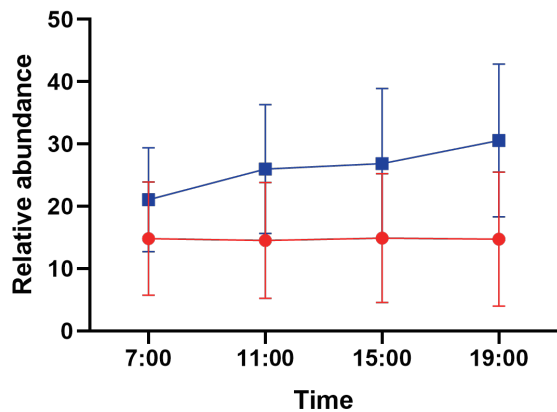**D****Fusobacteria**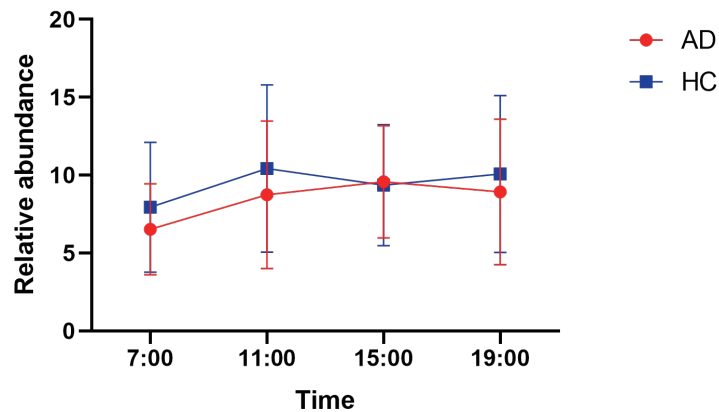

Supplement: Supplementary file 2 [file DataSheet_2.pdf]

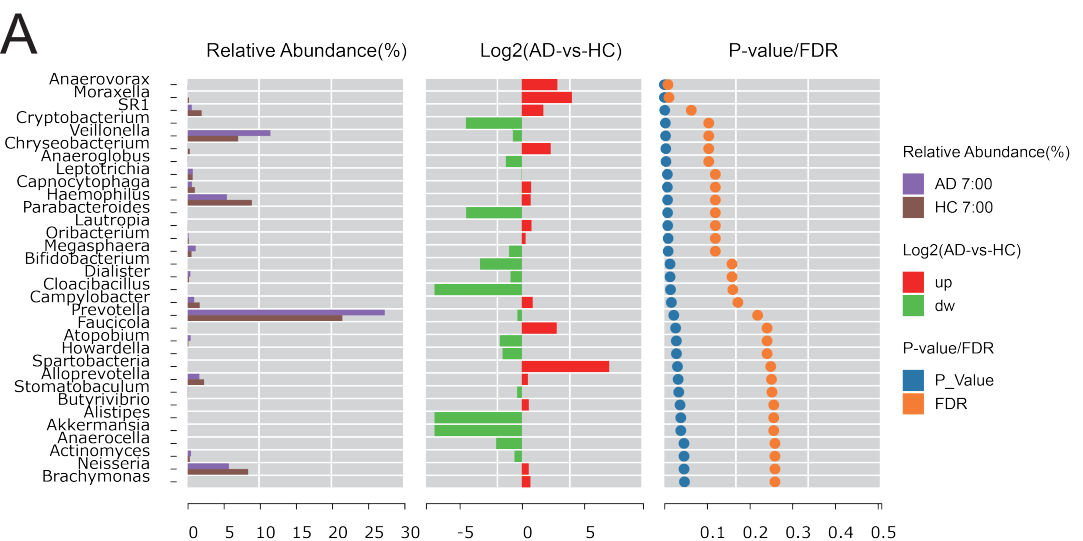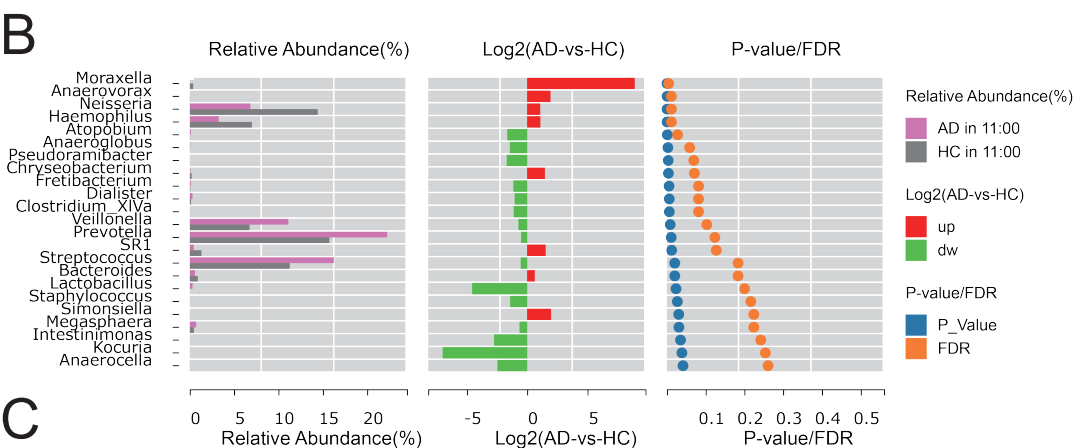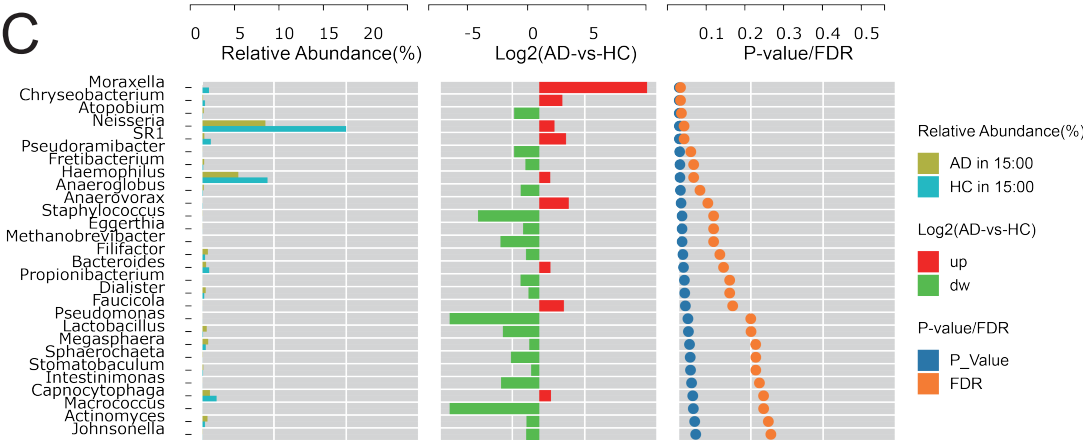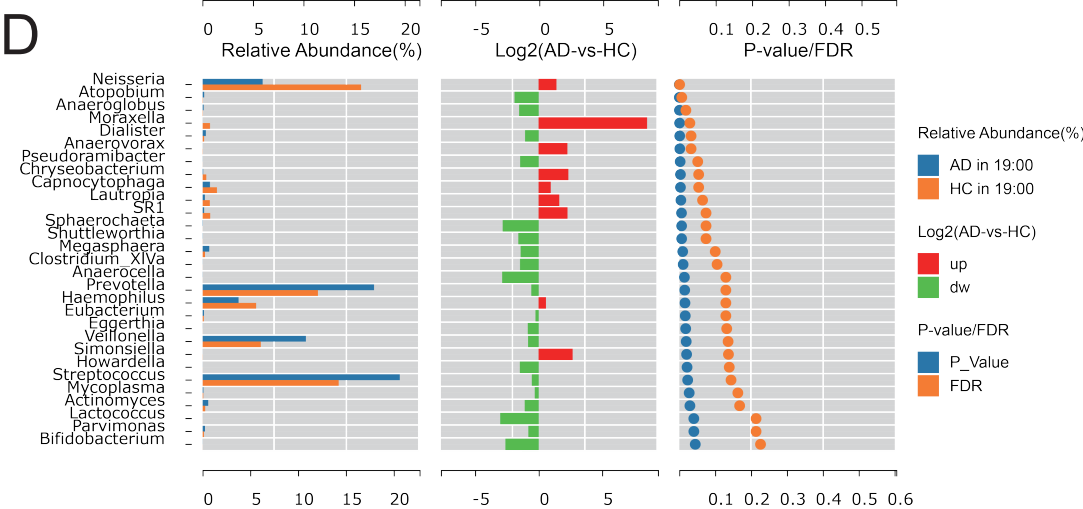

Supplement: Supplementary file 3 [file DataSheet_3.pdf]

A

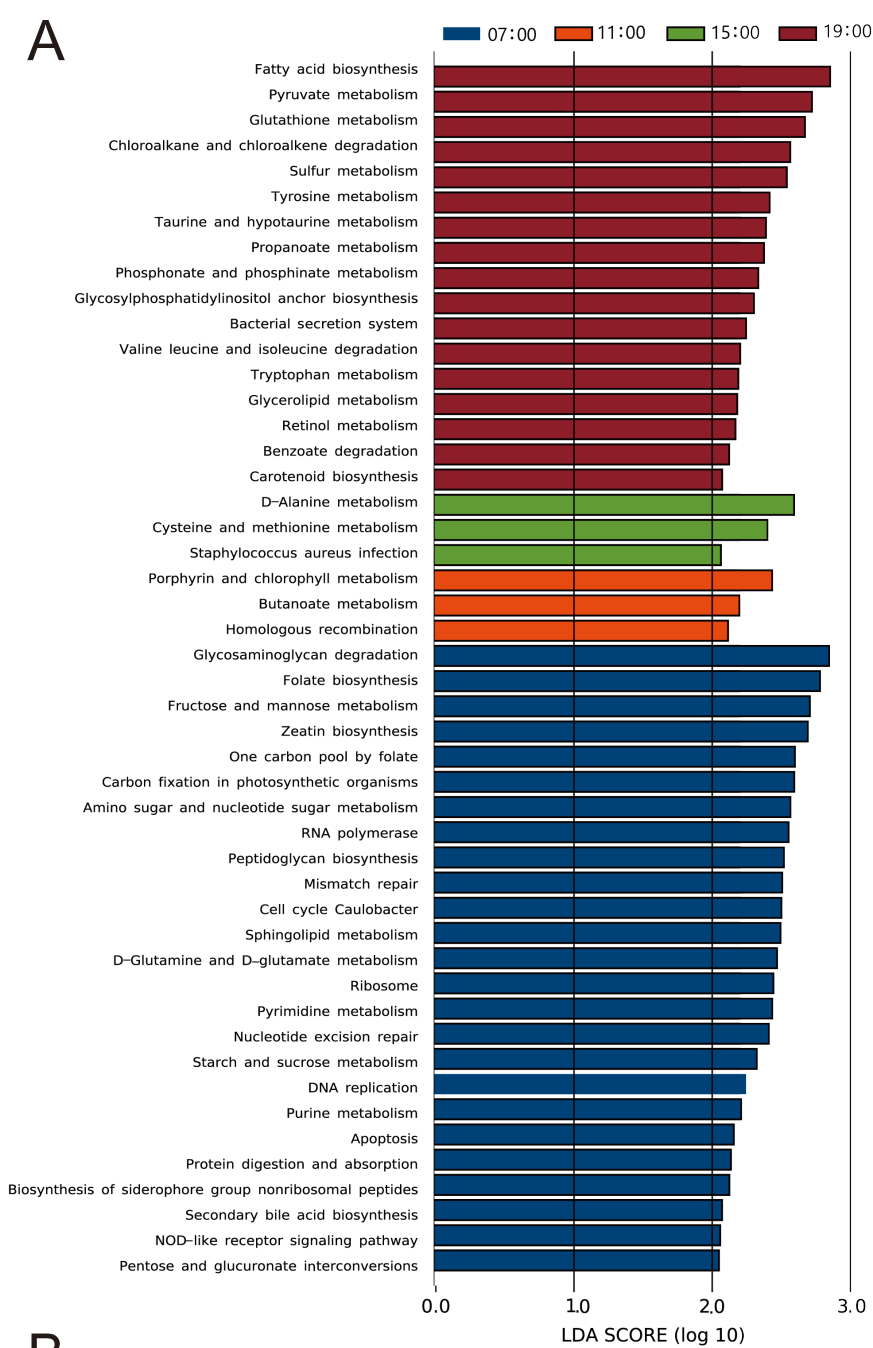

B

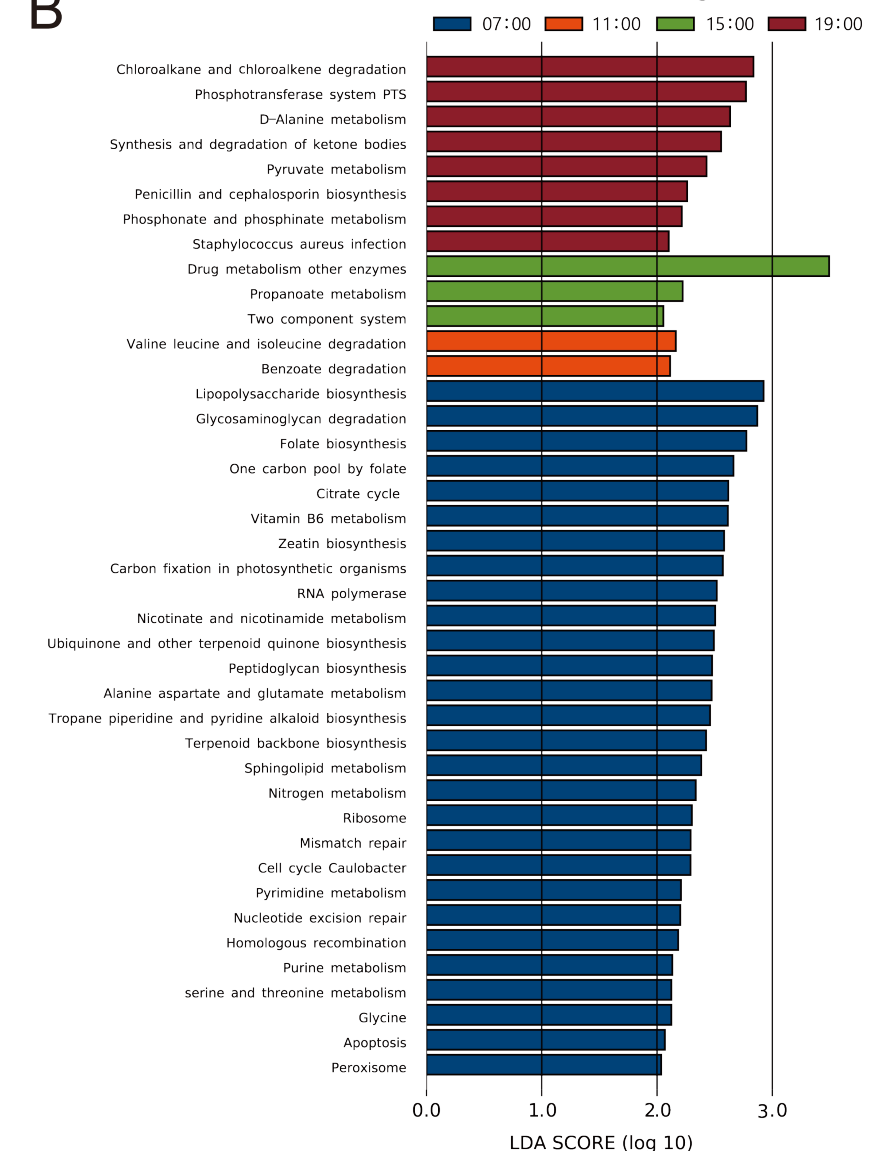

Supplement: Supplementary file 4 [file DataSheet_4.pdf]
