# Supplementary material for: Diurnal changes of the oral microbiome in patients with alcohol dependence
Source: Front Cell Infect Microbiol. 2022 Dec 12;12:1068908. doi: 10.3389/fcimb.2022.1068908 (PMC9791055; doi:10.3389/fcimb.2022.1068908)
Supplement: Supplementary file 6 [file Table_2.docx]

Aug 21, 2022 for *DRUG AND ALCOHOL DEPENDENCE*

***Supplemental Information***

**Diurnal Changes of the Oral Microbiome in Patients with Alcohol Dependence**

**Supplemental Material and Methods**

### Participants

The AD group enrollment criteria:

(1) Meet the DSM-IV diagnostic criteria for alcohol dependence;

(2) 18-60 years old, male;

(3) Han nationality.

The AD group exclusion criteria:

(1) Had or having infectious disease;

(2) Had or having heart, brain, liver, kidney and other serious diseases;

(3) Had or having metabolic diseases that can lead to abnormal immune system, such as obesity (BMI> 30 kg/m2), diabetes, rheumatoid arthritis, etc.;

(4) Had or having neurodegenerative diseases, such as Parkinson's disease;

(5) Any steroidal and non-steroidal anti-inflammatory drugs, antibiotics, antioxidants and immunosuppressive agents were used within 2 months before enrollment;

(6) Use probiotics and probiotics every day for the first 2 months before enrollment;

(7) Previous or current DSM-IV diagnosis of schizophrenia, depression, anxiety disorder, bipolar disorder, mental retardation, dementia (excluding mild cognitive function), and substance dependence other than alcohol and nicotine;

(8) Had irregular eating habits that affected oral flora (except alcohol) in recent 2 months;

(9) Abstinence from alcohol has exceeded 5 days;

(10) Having oral diseases.

The HC group enrollment criteria:

(1)18-60 years old, male;

(2) Han nationality.

The HC group exclusion criteria:

1. - (10) Exclusion criteria for patients with alcohol dependence, except (7) patients with a previous or current DSM-IV diagnosis of schizophrenia, depression, anxiety disorder, bipolar disorder, mental retardation, dementia (excluding MCI), and substance dependence, including alcohol (excluding nicotine); (12) Drinking alcohol during sample collection.

### Genomics DNA extraction

The microbial community DNA was extracted using the NEBnext microbiome DNA enrichment kit (New England Bio labs, Ipswich, MA, US) following the manufacturer's instructions. DNA was quantified with a Qubit Fluorometer by using a Qubit® dsDNA BR Assay kit (Invitrogen, USA), and the quality was checked by running an aliquot on a 1% agarose gel.

### Figure legends

Supplementary Figure 1. (A and B) Non-metric multidimensional scaling (NMDS) showed the rank order between the four groups and ANOISM test was preformed to quantify the difference in the AD and HC group.

Supplementary Figure 2. (A-D) The abundance of individual bacteria changes during a day time. Relative abundance of significant taxa in AD and HC conditions across time points: (A) Firmicutes, (B) Bacteroidetes, (C) Proteobacteria, (D) Fusobacteria.

Supplementary Figure 3. (A - D) The histogram of the relative abundance of each group is shown on the left. In the middle is the log2 value of the average relative abundance ratio of the same species in the two groups; the right figure shows the p-value and FDR values obtained by the Wilcox test. A. 7:00, B. 11:00, C.15:00, D.19:00.

Supplementary Figure 4. (A and B) LEfSe results showed a statistically significant increase in the abundance of KEGG pathways in the AD and HC groups. LEfSe results showed a sequentially significant ranking (P＜0.05) among classes (Kruskal-Wallis test) and between subclasses (Wilcoxon’s test). The threshold for the logarithmic LDA score was 2.0.
